# Computation of vertical fluid mobility of CO$_{2}$, methane, hydrogen and hydrocarbons through sandstones and carbonates

**DOI:** 10.1038/s41598-022-14234-6

**Published:** 2022-06-17

**Authors:** Bhavik Harish Lodhia, Stuart Raymond Clark

**Affiliations:** Department of Minerals and Energy Resources Engineering, University of New South Wales, Sydney, 2052 Australia

**Keywords:** Carbon capture and storage, Economic geology

## Abstract

Over the last decade, there has been an irreversible shift from hydrocarbon exploration towards carbon storage, low-carbon energy generation and hydrogen exploration. Whilst basin modelling techniques may be used to predict the migration of hydrocarbons through sedimentary basins on geological timescales, there remains little understanding of how fluids behave at the basin scale on present-day timescales. We apply the Darcy flow equation to present an algorithm to determine the basin-scale mobilities and maximum vertical velocity, $v_{max}$, of CO$_{2}$, methane, hydrogen and hydrocarbons with depth for sandstone and carbonate. $v_{max}$ for CO$_{2}$ and methane are on scales of m/year, whilst values for hydrocarbon fluids are an order of magnitude smaller than for other fluids. Our results indicate that the fluid mobility of subsurface CO$_{2}$ may be sensitive to surface and near-surface temperature variations. $v_{max}$ for hydrogen is approximately 2–10 times greater than hydrocarbon fluids, yielding important consequences for the future use of basin modelling software for determining hydrogen migration for exploration and storage.

## Introduction

The last decade has seen an irreversible shift in research focus away from hydrocarbon exploration and towards low-carbon energy production. This is reflected by the prioritisation of natural gas production over the use of high-CO$_{2}$ generating energy sources such as oil and coal, by many developed economies across the world (e.g. United States and Australia^1,2^). Shifting geopolitical climates around the world have also seen several countries (e.g., Denmark and United Kingdom) commit to achieving net-zero economies and power generation by 2050^3,4^. Many of the world’s largest energy producing companies have responded by committing to achieve carbon net-zero by 2050^5,6^. Hence, there is a growing interest across industry and the scientific community at applying well known techniques for hydrocarbon exploration to low-carbon technologies, such as carbon capture and storage (CCS) and green hydrogen exploration. Development of these techniques is dependent on understanding how CO$_{2}$, hydrogen and other fluids migrate through rocks. Traditionally, the history of fluid movement (e.g. hydrocarbons) through rocks across geologic time-scales has been calculated by basin modelling: a process by which sedimentary basins and their associated fluids are investigated to determine if the past conditions were appropriate to fill potential reservoirs with hydrocarbons and preserve the potential reservoirs^7^. The ability to simulate the passage of fluids through rocks is of significant importance to both hydrocarbon exploration and low-carbon activities, such as CCS. A large portion of processing time in basin modelling is used to solve Darcy flow equations in each grid cell of the model. The Darcy flow equation defines fluid velocity as

1 $$\begin{aligned} v_{max} = -\frac{k_{rock} k_{rp}}{\nu } |\nabla \mathbf{u } | \end{aligned}$$

whereby $k_{rock} =$ rock permeability, $k_{rp} =$ relative permeability, $\nu =$ fluid viscosity and $|\nabla \mathbf{u } |=$ driving force. The quantity $k_{rock}k_{rp} =$ effective permeability and $k_{rock}k_{rp}/\nu$ is defined as fluid mobility. As Eq. (1) does not account for direction, calculated velocities represent a maximum value ($v_{max}$) oriented in the direction of the driving force, $|\nabla \mathbf{u } |$. In the case of fluid flow in geological media, $|\nabla \mathbf{u } |=$ buoyancy:

2 $$\begin{aligned} |\nabla \mathbf{u } |= (\rho _{w} - \rho _{f})g \end{aligned}$$

whereby $\rho _{w} =$ density of water, $\rho _{f} =$ density of fluid and $g =$ gravity (9.80665 m/s$^{2}$)^7^. The mobility of a fluid is therefore a function of both fluid properties and rock type of the carrier, whereas buoyancy is independent of rock type. The terms of Eq. (1) include several important quantities that must be evaluated separately using the appropriate relationships that describe fluid volume, viscosity, density and buoyancy. A detailed description of the Darcy flow equation is described in “Methods” section.

Two- and three-dimensional basin models may take several days to compute on a home computer. This could be improved by assigning a fluid velocity that is dependent on properties of the carrier rock and depth. However, determining the velocities of fluids through different rock types remains a significant challenge. The overwhelming majority of research on fluid flow through rocks is focused on laboratory studies of specific examples or case studies or unsuccessful application of basin modelling software to simulate real-time fluid flow^8^. As we shall see, the determination of fluid velocities in different rock types as function of depth will be of immense value to both basin and future modelling studies for hydrocarbon exploration, CCS and potentially green hydrogen exploration.

In this study, we apply various Equations of State and bulk properties of common rock types to different fluids encountered within the subsurface to calculate the variation of physical properties (e.g. volume and viscosity) with depth and surface temperature. Depositional parameter values for two generalised rock types, sandstone and carbonate, are applied in this study. For carbonate, parameter values for micrite are used. Depositional porosity ($\phi _{0}$) values are 0.41 and 0.51 and compaction wavelengths are 3.22 km and 1.92 km for sandstone and carbonate, respectively^7^.

We present an algorithm to calculate maximum vertical velocity, $v_{max}$, as a function of fluid mobility and buoyancy using the Darcy flow equation (Eq. 1). The results of this study may have important implications for the application of basin modelling techniques to understand migration of non-hydrocarbon fluids and for threshold depths, below which fluids have low mobilities.

**Figure 1:**
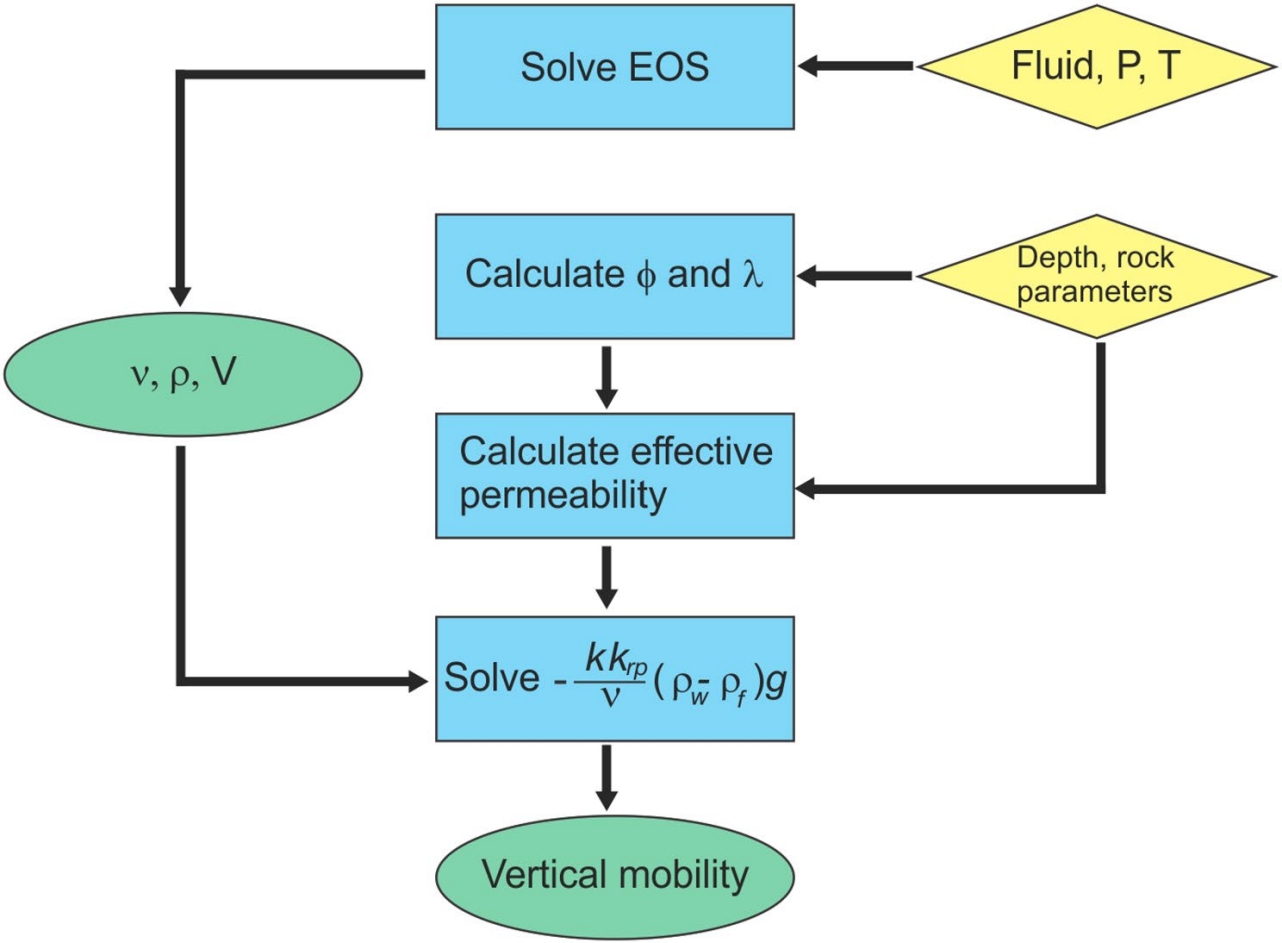
Methodology to calculate vertical mobility of fluids. Diamonds = inputs, rectangles = processes, ovals = outputs.

Using Athy’s relation and the values in Tables 1 and 2, we use the following algorithm to calculate the vertical mobility and buoyancy of CO$_{2}$ and methane (outlined in Fig. 1):

3 $$\begin{aligned} \begin{array}{ll} (1) &{} {\text{ Solve } \text{ chemical } \text{ equation } \text{ of } \text{ state } \text{(EoS) } \text{ to } \text{ calculate } \text{ fluid } \text{ volume } \text{ from } \text{ pressure } \text{ and } \text{ temperature }} \\ (2) &{} {\text{ Calculate } \text{ rock } \text{ porosity } } (\phi ) { \text{ and } \text{ permeability } \text{ at } \text{ depth }} \\ (3) &{} {\text{ Calculate } \text{ fluid--water } \text{ relative } \text{ permeability } } (k_{rp}) { \text{ and } \text{ connate } \text{ water } \text{ saturation } } (S_{w}) { \text{ from } \text{ porosity }} \\ (4) &{} {\text{ Calculate } \text{ fluid } \text{ viscosity } \text{ from } \text{ pressure } \text{ and } \text{ temperature }} \\ (5) &{} {\text{ Calculate } \text{ vertical } \text{ fluid } \text{ mobility } \text{ and } \text{ buoyancy }} \\ \end{array} \end{aligned}$$

**Table 1:** Component properties of hydrocarbon gases, hydrogen, CO$_{2}$ and water^7,9–11^.

	M$_{\text{ w }}$ (g/mol)	$T_{c}$ ($^{\circ }$K)	$P_{c}$ (MPa)	$v_{c}$ (m$^{3}$/kmol)	$\omega$
CO$_{2}$	44.010	304.19	7.382	0.0940	0.2276
Methane	16.043	190.56	4.599	0.0985	0.0115
Dry gas	17.943	197.42	4.850	0.0977	0.0221
Wet gas	30.186	272.40	4.801	0.1345	0.0624
H$_{2}$	2.016	33.18	1.313	0.0642	-0.215
Volatile oil	53.135	367.79	4.372	0.1956	0.1267
Light oil	48.439	357.38	4.389	0.1895	0.1238
Black oil	90.072	491.62	3.535	0.2423	0.3121
Water	18.015	647.13	22.055	0.0560	0.3449

$M_{W}$ = molecular weight, $T_{C}$ = critical temperature, $P_{c}$ = critical pressure, $v_{c}$ = critical volume, $\omega$ = acentric factor.

**Table 2:** Rock properties.

Name	$a_{k}$	Porosity at point			Permeability [logmD] at point			Athy *k* (km)	Athy *k* (MPa)
		1	2	$\phi _{0}$	1	2	$\phi _{0}$		
Sandstone	5	0.01	0.25	0.41	− 1.8	3	4.33	3.225	37.593
Carbonate	1.1	0.01	0.25	0.51	− 2.20	1.00	1.52	1.923	20.982

Anisotropy = $a_{k}$. Porosity and rock permeability values used to generate multipoint curves and rock saturation endpoint values for bulk rock. $\phi _{0}$ = maximum (depositional) porosity of rock^7^. These values were used to determine the rock permeability from porosity shown on Fig. 8.

## Results

**Figure 2:**
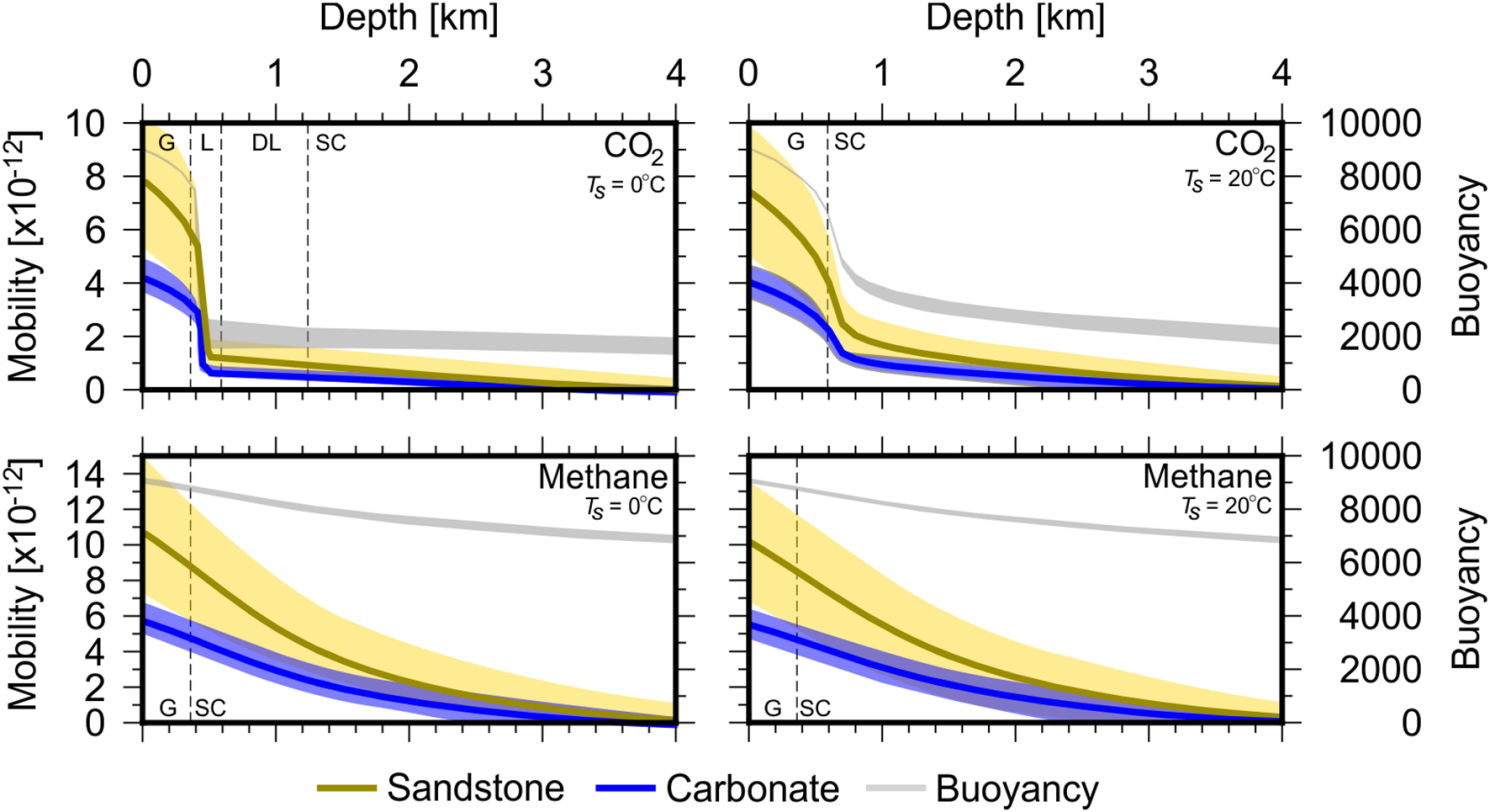
Fluid mobility for CO$_{2}$ and methane calculated according to Eq. (1). Fluid mobilities are calculated at $T_{s} = 0\,^{\circ }$C (left column) and $T_{s} = 20\,^{\circ }$C’. (right column), respectively. Units for fluid mobility are m$^{2}$ Pa$^{-1}$ S$^{-1}$ and buoyancy are kg m$^{-2}$ S$^{-2}$. Yellow and blue lines represent fluid mobility in sandstone and carbonate, respectively. Grey lines represent buoyancy and shaded regions represent range of values calculated using different EoS and lithological parameters for Eq. (14). Dashed lines represent phase transitions: *G* gas, *L* liquid, *DL* dense liquid and *SC* super-critical.

The results for fluid mobility and buoyancy calculations for CO$_{2}$ and methane at normal geological conditions for surface temperatures of 0 $^{\circ }$C and 20 $^{\circ }$C are shown on Fig. 2. Gas phase mobility increases exponentially as depth decreases, with maximum mobility at the surface. Non-gas phase mobilities for both CO$_{2}$ and methane in both sandstones and carbonates are significantly lower than corresponding gas phase mobilities and decrease exponentially with depth. Fluid buoyancy follows a similar pattern.

The choice of EoS has a minimal impact on calculated fluid mobility and buoyancy for CO$_{2}$ and methane. Fluid buoyancies increase exponentially upon the transition to gas phase. Methane mobility varies little with $T_{s}$, with results indistinguishable for $T_{s} =0\,^{\circ }$C and 20 $^{\circ }$C. Calculated vertical fluid mobilities for CO$_{2}$ are particularly sensitive to $T_{s}$. Fluid mobilities for both CO$_{2}$ and methane are approximately double in sandstone compared to carbonate. Whilst the magnitude of vertical mobilities of fluids vary little with surface temperature, the depth of liquid–gas phase transitions vary considerably where critical properties are close to surface conditions (Fig. 7).

**Figure 3:**
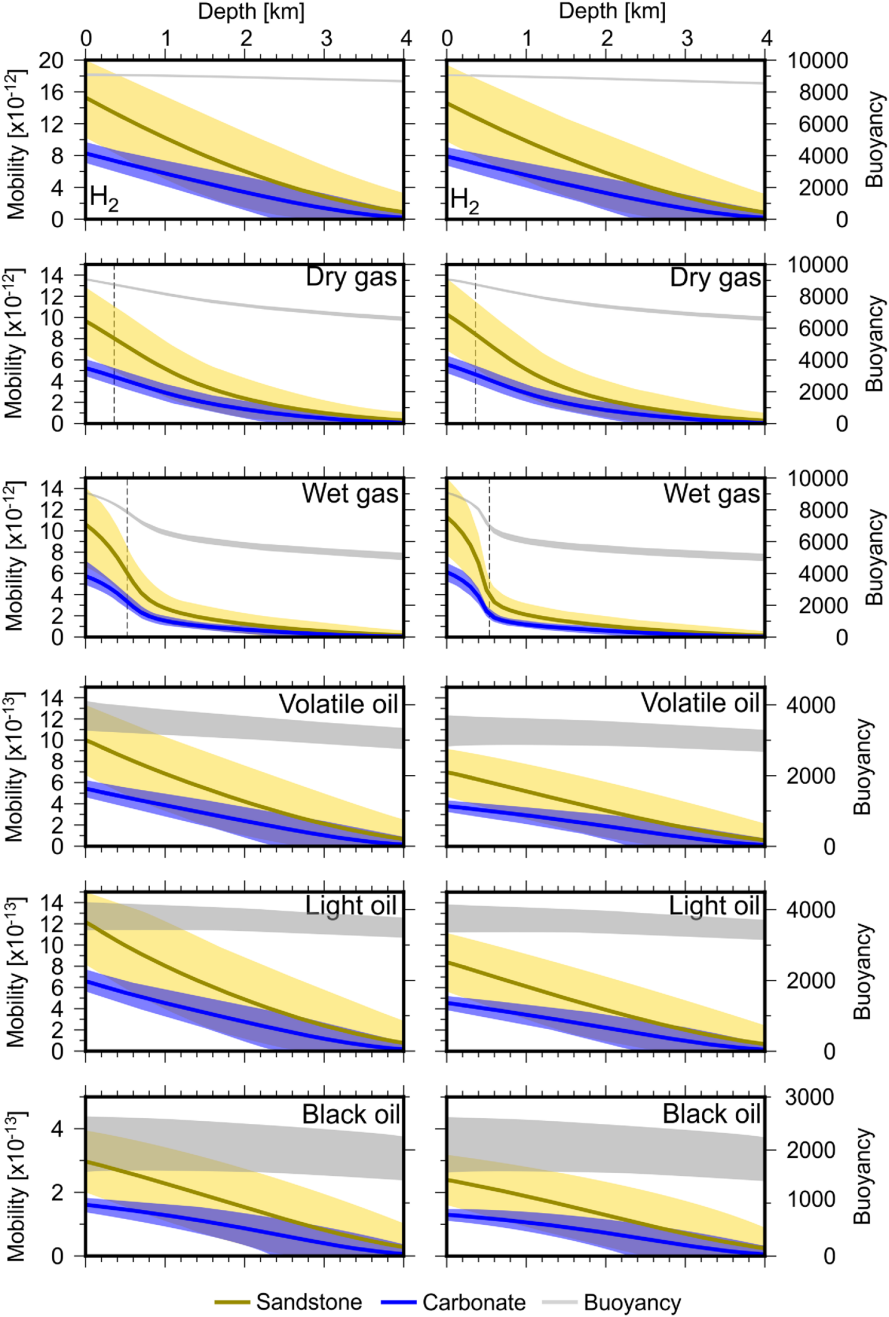
Fluid mobility for non-hydrocarbon and hydrocarbon fluids listed in Table 1 calculated according to Eq. (1). Units for fluid mobility are m$^{2}$ Pa$^{-1}$ S$^{-1}$ and buoyancy are kg m$^{-2}$ S$^{-2}$. Yellow and blue lines represent fluid mobility in sandstone and carbonate, respectively. Grey lines represent buoyancy and shaded regions represent error. Wet and dry gas exhibit similar properties to CO$_{2}$ and methane, respectively. Phase transitions for CO$_{2}$ and methane are plotted on wet and dry gas, respectively as dashed lines.

**Figure 4:**
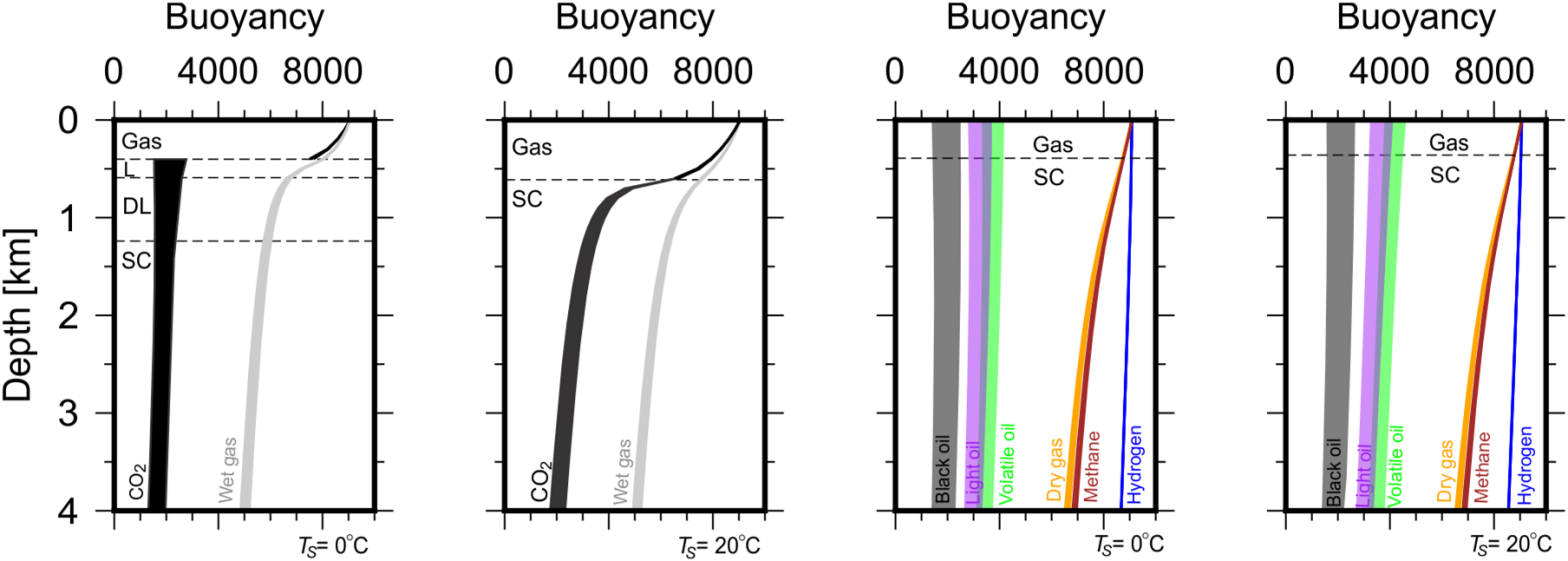
Fluid buoyancy calculated for other hydrocarbon and non-hydrocarbon fluids listed on Table 1 calculated using PR78, SRK and RK EoS for dry/wet gas and hydrogen and using appropriate EoS for hydrocarbon fluids^12–19^. Wet gas exhibits similar properties to CO$_{2}$ at $T_{s} = 20\,^{\circ }$C, whilst dry gas exhibits similar properties to methane. Hydrocarbon fluid buoyancies are significantly lower than dry gas and methane and decrease with molecular weight. Hydrogen buoyancy varies little with depth.

The equations used to relate relative permeability and normalised water saturation (Eq. 13) are designed for application to fluid systems with water, oil–water and gas–oil components. However, it is intriguing to extend this analysis to other fluids. The properties of several other important hydrocarbon fluids and hydrogen are listed in Table 1. We apply the algorithm proposed in this study to calculate vertical fluid mobility and buoyancy for hydrogen, dry/wet gas and various hydrocarbon fluids (Figs. 3 and 4).

Unlike for CO$_{2}$, the vertical fluid mobilities and buoyancies of all other fluids considered in this study do not show significant sensitivity to $T_{s}$. The magnitude of vertical fluid mobilities of hydrogen are up to twice those of dry/wet gas and over an order of magnitude greater than other hydrocarbon fluids (volatile, light and black oil). The buoyancy of hydrogen varies little with depth as all conditions within the Earth are beyond its critical parameters. Whilst above 0.36 km, the buoyancy of hydrogen is close to that of other gas phase fluids (methane, dry and wet gas, Fig. 4) and is $>\,8000$ kg m$^{-2}$ S$^{-2}$. Hence, this raises the important consideration that hydrogen velocities may be 2–10 times greater than those for other fluids and must be accounted for when evaluating hydrogen migration whilst applying conventional fluid flow modelling techniques suited for hydrocarbon migration. The vertical fluid mobilities for volatile, light and black oils are an order of magnitude smaller than those for other fluids, reflecting the fact that these remain in the liquid phase at all levels below the surface. Whilst results for dry gas are close to those for methane, results for wet gas resemble the pattern seen for CO$_{2}$ when $T_{s} = 20\,^{\circ }$C. This is expected, as conditions within the Earth exceed the critical properties of wet gas (Table 1).

## Discussion

The results of this study have several important implications. The velocity of fluids through rocks may be calculated directly by multiplying vertical fluid mobility and buoyancy, according to Eq. (1). Results indicate maximum vertical gas-phase velocities of 1.5–4 m/year (sandstone) and 1–2.5 m/year (carbonate) for CO$_{2}$ and methane, respectively. As both vertical fluid mobility and buoyancy decrease exponentially below the gas-supercritical or gas–liquid phase transition, our results indicate a minimum depth for CO$_{2}$ storage 0.36 km and 0.59 km when $T_{s} = 0\,^{\circ }$C and 20 $^{\circ }$C, respectively.

### Basin modelling

Modelling of CO$_{2}$ and H$_{2}$ migration are dependent on understanding the present-day velocities of fluids. However, there exists no direct method for calculating fluid velocities as a function of lithology and depth. Attempts to apply basin modelling software to predict the effect of fluid injection on basin-scale pressure evolution have been largely unsuccessful^8^. Whilst the assumptions used in this work are relevant for water, oil or gas fluid systems in generalised sandstones and carbonates, our results could have important implications for future investigations for carbon storage and hydrogen exploration.

**Figure 5:**
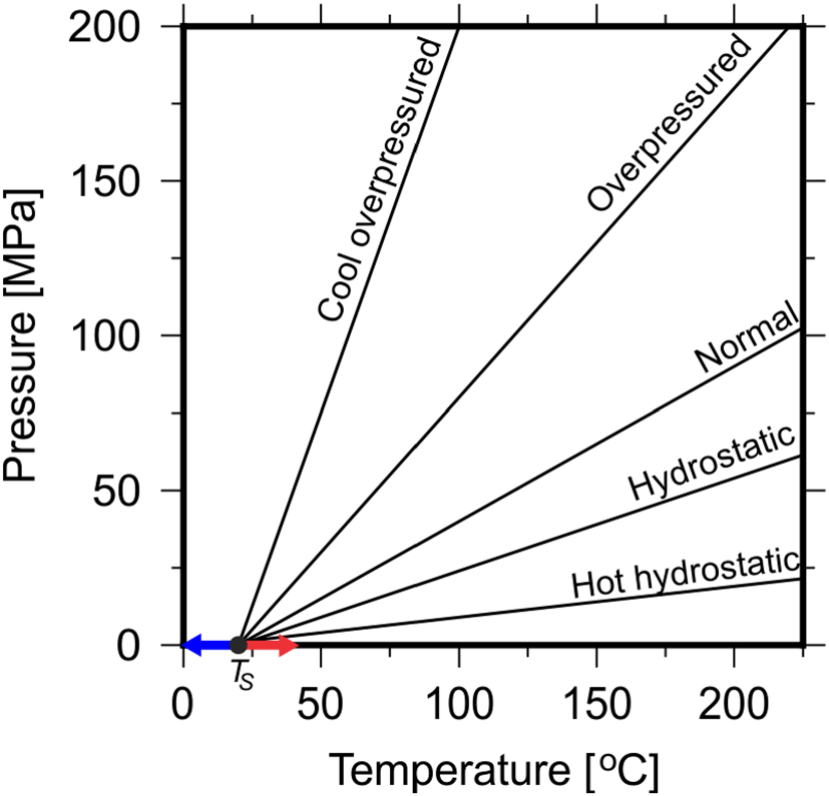
Typical pressure temperature (PT) paths in geological basins with a surface temperature of $20\,^{\circ }$C^7^. PT gradients are as follows: cool and overpressured = 2.5 MPa/K, overpressured = 1.0 MPa/K, normal = 0.5 MPa/K, hydrostatic = 0.3 MPa/K and hot hydrostatic = 0.1 MPa/K.

Sedimentary basins around the world are often characterised by complex geological histories. The PT-path of sedimentary basins will likely cross several regimes (e.g. different lines on Fig. 5). The methodology outlined in this study may be used to evaluate fluid velocities through different geological regimes (e.g. moving from normal conditions to regions of overpressure) by choosing the appropriate PT-path for discrete depth intervals. The methodology presented in this study, if combined with a directional vector, may provide a fast and computationally inexpensive means to calculate fluid velocities as a function of depth and angle of incidence between fluid particles and geological horizons, e.g. impermeable seals. A potential method to calculate the direction of fluid flow beneath geological seals is flowpath bending, which is widely used to model petroleum flow in basin modelling software. A planar seal with dipping angle $\alpha$ in the y-direction and lateral water flow with angle $\beta$ to the x-axis and a hydraulic head with a dipping angle of $\gamma$ are used to define the angle $\psi$, which indicates the direction of fluid flow as:

4 $$\begin{aligned} tan \psi = tan \beta cos^{2}\alpha \frac{\Delta \rho }{\rho _{w}} \frac{cos \alpha sin \alpha }{tan \gamma cos \beta } \end{aligned}$$

whereby $\Delta \rho =$ difference in density between fluid and water and $\rho _{w} =$ water density^7^. When water flow is also assumed to follow the dipping of the seal, this becomes:

5 $$\begin{aligned} tan \psi = tan \beta \frac{\Delta \rho }{\rho _{w}}\sqrt{1 + sin^{2}\beta tan^{2}\alpha }\frac{cos \alpha sin \alpha }{tan \gamma cos \beta } \end{aligned}$$

Combining calculations for $v_{max}$ and directional vectors could significantly reduce fluid flow simulation processing time compared to leading basin modelling software packages that rely on the creation of nodal meshes to process fluid particle-rock interactions.

The range of uncertainties for $S_{w}$ becomes large beneath depths of 2 km (Fig. 9). Future work should focus on expanding our understanding about the relationship between porosity and water saturation in different rock types. New experimental data for a wide range of rock types will improve Eq. (14) and broaden the possibilities for the methodology described in this study.

It is widely accepted that hydrocarbons have the capability to migrate long distance through carriers on geological timescales. For example, Hantschel and Kauerauf^7^ estimate the velocity of oil with density $=\,400$ kg m$^{-3}$ in carriers with $k_{rock}k_{rp} \sim \, 1$ mD $\sim \,10^{-15}$ m$^{2}$ as $v \sim 6$ nm s$^{-1}$, equivalent to $\sim \,180$ km Ma$^{-1}$. Using values for sandstones, volatile and light oils, our results indicate vertical mobilities of 14–6 $\times 10^{-13}\,$m$^{2}$ Pa$^{-1}$ S$^{-1}$ and buoyancies of 3700–1700 kg m$^{-2}$ S$^{-2}$ between depths of 0 and 2 km. These yield values yield vertical velocity values of $\sim$ 5.2–1.0 nm S$^{-1}$, or $\sim$ 164–32 km Ma$^{-1}$. The results of this work indicate that CO$_{2}$, methane, dry gas and wet gas may migrate several metres vertically per year ($>\,1000$ km/Ma) when in the gas phase. It is very encouraging that these values are consistent in magnitude with reports in the literature of hydrocarbon velocities of up to 1000 km/Ma, and indicate that the approximations used in this method are appropriate^20^. It is also encouraging that exponential increases in fluid velocities are concomitant with liquid–gas phase transitions given that this is independent of rock type. Furthermore, the magnitudes of vertical mobilities match those reported for hydrocarbons of fluids of similar composition and molecular weights to those analysed in this study (excluding H$_{2}$).

Whilst the Darcy flow equation (Eq. 1) is widely accepted as the leading method to describe fluid flow in porous media, there are several important drawbacks. Averaging of microscopic features, such as discontinuities, holes, fractures and microporosities allows for upsaling to macroscopic length scales. However, a disadvantage of this method is the resulting low spatial resolution which constricts modelling of migration channels. Basin models typically cover large areas ($10 \times 10$ km to $1000 \times 1000$ km) and are gridded and layered. Up to 500 grid points and 50 layers may be specified, depending on model complexity. Volume elements within each layer contain a constant facies bulk continuum approximation and treat all elements within a volume as constant^7^. Upscaling of physical properties from the core to grid size is necessary to capture the effects of physical features characteristic of specific lithologies. This is accounted for in part by applying appropriate upscaling factors. However, upscaling parameters may be amended as necessary for different rock types (e.g. using laboratory data) to improve overall model robustness in future studies.

### Carbon storage and methane

The geological storage of CO$_{2}$ is dependent on the existence conditions that keep CO$_{2}$ in the liquid or supercirical phase and has been a subject of significant interest over the last three decades. Long-term storage of CO$_{2}$ under the ground at the subsurface is considered feasible for three types of geological formations: hydrocarbon reservoirs (depleted), deep saline aquifers and unminable coal formations. When considering potential sites for CO$_{2}$ storage, various geological characteristics must be taken into account, including characterising cap rocks to determine the effectiveness of a seal, if there are any abandoned or active wells which can compromise seal integrity and whether there is a sufficiently large and permeable storage formation^21,22^. With low levels of radiogenic heat production, shallow sandstone and carbonate layers within onshore sedimentary basins will typically exhibit lower pressures and temperatures compared to offshore depleted hydrocarbon reservoirs. However, the nature and the effects of surface and near-surface temperature variations on the physical properties of subsurface pore fluid–CO$_{2}$ systems remain relatively unexplored.

CO$_{2}$ phase behaviour is primarily controlled by subsurface temperature and pressure conditions. Time series of subsurface temperatures and records of surface air temperatures indicate that subsurface temperatures are attenuated by several degrees up to depths of 10 m. However, intrinsic rocks are not influenced beyond this depth. For example, in warm climates the temperature 1 m below the ground surface remains below $30\,^{\circ }$C whilst the ground surface temperature may reach $65\,^{\circ }$C^23,24^. However, fluids transported via pathways created by heterogeneities intrinsic to rock volumes and fractures (e.g. faults), may play an important role in transporting heat from the surface to rocks at depth^25^. Low-frequency temperature signals (e.g. decadal climate change) may be retained at depth ($>100$ m) while high-frequency temperature signals (e.g. monthly) are generally retained at shallow depths ($<10$ m)^26^. Recent research indicates that fluids migrating along fracture networks through rock volumes may be significant propagators of heat, and the combined effects of urbanisation and global warming may reach more than 100 m below the surface^27–29^. Taniguchi et al.^28^ measure the depth of deviation from regional geothermal gradients in several major Asian cities, with deepest occurring in Tokyo, at 140 m. Furthermore, recent investigations into the effects of large-magnitude urban heat islands indicate the creation of significant downward components of conductive heat flow in the shallow subsurface, which are supplemented by downward heat transport by groundwater movement^29^. Hence, elevated groundwater temperatures may have potential consequences on the state of CO$_{2}$ hundreds of metres beneath Earth’s surface. As shown in Fig. 7, the pressure required to cross the CO$_{2}$ supercritical phase boundary increases with temperature. With a lack of corresponding increases in pressure, increases in temperature due to heat transportation from groundwater may cause CO$_{2}$ at depth to remain in the gaseous phase. In the absence of elevated subsurface temperature (e.g., from urban heat islands, igneous activity etc.), our results indicate that the depth of the CO$_{2}$ liquid–gas phase boundary changes from 0.36 to 0.59 km between surface temperatures of $0\,^{\circ }$C and $20\,^{\circ }$C, respectively. We interpret this to mean that CO$_{2}$ remains stable in a liquid form at shallower depths beneath regions of lower surface temperature. Therefore, it is our opinion that the long-term efficiency of CO$_{2}$ injection will be maximised at northern latitudes in regions not affected by urbanisation, igneous activity or other sources of increased subsurface heat flow. It is intriguing that changes surface and near-surface temperatures due to urbanisation and climate change over the last several centuries may affect the geological storage potential for CO$_{2}$, and further research is required in this area.

The application of PT paths (Fig. 5) and EoS to determine fluid mobilities assume steady-state basin conditions and is appropriate for modelling past fluid migration during basin evolution, e.g., basin modelling. However, during CO$_{2}$ injection, forcing terms (i.e., inflow and outflow) must be considered to evaluate fluid properties at the injection site and surroundings. Whilst it is beyond the scope of this study to consider these in our algorithm, it is reasonable to imply that the algorithm proposed may be of use to model CO$_{2}$ migration within geological basins post-injection, over both human and geological timescales.

One limitation is that the cubic EoS applied in this study neglect fluid dissolution in water. It is commonly assumed that hydrocarbons do not dissolve in water, with the exception of methane. In the subsurface, methane dissolves in water only in the immediate neighbourhood of hydrocarbon pathways. Methane dissolution can be treated separately from hydrocarbons and is dependent on the amount of methane, pressure and temperature^30^. Another important consideration is that the physical properties of CO$_{2}$ differ from typical hydrocarbons. CO$_{2}$ is slightly polar and and dissolves well in water. Recent adaptations of cubic EoS (e.g., PR, SRK) have improved the accuracy of CO$_{2}$–CH$_{4}$–H$_{2}$O systems significantly by including an association term named the Cubic Plus Association, or CPA factor. Results from mixing experiments of CO$_{2}$, CH$_{4}$ and H$_{2}$O gases, CO$_{2}$-brine and CO$_{2}$–acid gas–H$_{2}$O systems show that in general, water content increases isobarically with temperature, decreases isothermally with pressure and increases with increased CO$_{2}$ concentration in the feed gas^31–34^. To explore the effects of surface and near-surface temperatures on groundwater–CO$_{2}$ and pore fluid–CO$_{2}$ mixtures in the subsurface, future research should focus on incorporating adaptations of cubic EoS and the application of rock properties for specific lithologies into the fluid mobility algorithm proposed in this study.

Gilmore et al.^35^ evaluate the case of a fault cutting through three vertically stacked aquifers and seals based on data from a naturally CO$_{2}$-charged aquifer at Green River, Utah. CO$_{2}$ has been escaping at the site along fault zones for several hundred thousand years. Their analyses indicates that despite leakage, the majority of trapped CO$_{2}$ remains stable when the fault permeability is is comparable or less than reservoir permeability. When fault permeability exceeds reservoir permeability, $> 50\%$ of injected CO$_{2}$ remains trapped after 1000 years. In reality, the ability for CO$_{2}$ and other fluids to migrate along faults depends on the relative differences in permeabilities between layers and faults, and will be determined by the geological complexity of the area. To determine migration pathways of CO$_{2}$ through multiple layers in geological basins, calculations of fluid mobility must be combined with adequate evaluation of carrier and fault permeabilities. Whilst the presence of impermeable geological seals and trapping mechanisms are of paramount importance during selection of CO$_{2}$ storage sites, risk of undetected fluid pathways capable of transporting groundwater into sedimentary rocks cannot be ignored. Examples of complex and vast systems of small-scale polygonal faults are likely to be responsible for fluid migration in post-rift sedimentary successions that were previously thought to be absent of migration pathways, e.g. Qiongdongnan Basin, China^36^.

We believe that our algorithm offers an important starting point to evaluate CO$_{2}$ mobility (and potentially velocity) using a computationally inexpensive method which accounts for gas–liquid and gas-supercritical phase changes with depth. The principles of the algorithm proposed in this study also offers a potential means to bridge the knowledge gap between modelling of CO$_{2}$ plume migration, rock properties and including the crucial step of an EoS which may be modified to account for polar fluid–water interactions. It is our hope that this initial study leads on to further research in this area and development of methods capable of calculating the effects of surface temperatures on CO$_{2}$ velocities during and after CO$_{2}$ injection for storage purposes.

### Hydrogen exploration

Momentum behind hydrogen exploration is growing, with jurisdictions such as South Australia granting or receiving applications for 18 exploration licenses by six different companies searching for natural hydrogen since February 2021^37^. Recent studies link the geological stratigraphic accumulation of hydrogen to the presence of shallow ($<10$ km) mantle rocks multi overlaid doleritic sills and aquifers^38,39^. Lefeuvre et al.^38^ show that major faults may act as conduits for natural hydrogen and provide a migration pathway from shallow mantle rocks under the Mauléon Basin, Western Pyrenees, to the surface. An example of a major natural hydrogen discovery and implementation of reservoirs is in Mali, west Africa. In 2012, natural hydrogen was discovered and eventually connected to a fuel cell to supply electricity to the town of Bourabougu^37,39^.

Hydrogen is generally considered insoluble in water below pressures of 40 MPa, with the Peng-Robinson and SRK EoS decreasing in accuracy as pressure increases^40^. When the hydrostatic pressure gradient for any region is approximately $\sim 10.5$ kPa/m, it is known as the normal pressure gradient. Abnormally high hydrostatic pressure gradients of $\sim 21.5$ kPa/m have been encountered, e.g., in geopressured/geothermal zones, Gulf of Mexico, Niger Delta and North Sea^41,42^. Abnormally low hydrostatic pressure gradients $<10.5$ kPa/m have also been encountered (e.g. Pennsylvania, Oklahoma^40^). Using values for the normal pressure gradient, it is reasonable to assume that the EoS applied in this study are accurate for modelling insoluble hydrogen properties to a depth of $\sim 1760$ m in most circumstances.

Clearly, the application of well known basin modelling techniques presents an attractive opportunity for hydrogen exploration. Application of the algorithm proposed in this study to model hydrogen migration at greater depths or in regions of high pressure may be possible with future research that focuses on amending widely used EoS to account for hydrogen solubility at pressures $>40$ MPa. Our results indicate that future application of basin modelling techniques and software for assessment of short-term basin-scale hydrogen storage should account for fluid mobilities that are 2–10 times greater than those of hydrocarbons at the same geological conditions above depths of $\sim 1760$ m where EoS remain stable.

## Conclusions

We present an algorithm to calculate vertical fluid mobility and buoyancy for CO$_{2}$ and methane as a function of depth for generic sandstone and carbonate by combining solutions to chemical equations of state, viscosity and permeability calculations and the Darcy-Flow equation. Vertical fluid mobility and buoyancy in both sandstone and carbonate decrease exponentially with depth and are significantly greater for gas phases compared to liquid, dense liquid or supercritical phases. The depth of the phase transitions for CO$_{2}$ is sensitive to surface temperature, whereby gas–liquid and gas-supercritical transitions occur at 0.36 km for $T_{s} = 0\,^{\circ }$C and 0.59 km for $T_{s} = 20\,^{\circ }$C, respectively. Surface temperatures $\,>0\,^{\circ }$C push the pressure-temperature profile of CO$_{2}$ into the gas-supercritical region. Hydrogen velocities may be approximately 2–10 times greater than those of CO$_{2}$, methane, dry and wet gas and over an order of magnitude greater than for other hydrocarbon fluids. Dry and wet gas follow similar trajectories as methane and CO$_{2}$. Vertical fluid mobility and buoyancy of other hydrocarbon fluids (volatile, light and black oil) are an order of magnitude smaller than those of CO$_{2}$, methane and dry/wet gas.

## Methods

### Darcy flow equation

As described in Hantschel and Kauerauf^7^, the driving forces for fluid flow are pressure potential differences, where the fluid pressure potential is the pressure reduced by the pressure of a static fluid column with a corresponding vertical pressure gradient. The potential, $u_{p}$ for any phase *p* is thus defined as:

6 $$\begin{aligned} u_{p} = p - \rho _{p} gz \end{aligned}$$

with $\rho _{p} =$ density of fluid phase *p*, $g =$ gravitational acceleration and $z =$ depth. Darcy’s law states that a potential difference causes flow according to:

7 $$\begin{aligned} v_{p} = \frac{\mu _{p} \Delta u_{p}}{\Delta l} \end{aligned}$$

Here, $v_{p}$ = velocity of phase *p* and $\mu _{p} =$ mobility. The mobility of in multi-phase fluid systems is usually split into three factors, such that:

8 $$\begin{aligned} \mu _{p} = \frac{k_{rock} k_{rp}}{\nu _{p}} \end{aligned}$$

where $k_{rock} =$ rock permeability, $k_{rp}$ = relative permeability and $\nu _{p} =$ viscosity of phase *p*. This provides a basic formulation of Darcy’s law, with a comprehensive formulation given by:

9 $$\begin{aligned} \varvec{v_{p} = - \mu _{p} \cdot \nabla u_{p}} \end{aligned}$$

The driving force $\varvec{-\nabla u_{p}}$ is a gradient, which points in the direction of the steepest decrease of the potential field $u_{p}$. It is multiplied with a tensor $\varvec{\mu _{p} \propto k}$ which describes the anisotropy of the rock permeability so that the resulting flow velocity $\varvec{v_{p}}$ is not necessarily pointing in the same direction as $\varvec{-\nabla u_{p}}$. The Darcy flow equation may therefore be evaluated as:

10 $$\begin{aligned} v= \frac{k_{rock} k_{rp}}{\nu } \cdot |\nabla u|\end{aligned}$$

In the case of fluid flow in the subsurface, the driving force $|\nabla u|$ is buoyancy between fluid phase *p* and water that is given by:

11 $$\begin{aligned} |\nabla u |= \left( \rho _{w} - \rho _{p}\right) g \end{aligned}$$

### Fluid properties in rocks

Fluids may be grouped into compounds with approximately the same physical properties, and are typically ‘pure’ (e.g,. CO$_{2}$, methane or water) or ‘lumped’ chemical species (e.g., alkanes)^9^. The subject of fluid analysis can be subdivided into three parts, the determination of the coexisting phases, their compositions and their properties. In this study, we consider both pure fluids (CO$_{2}$, methane, and hydrogen) and lumped fluids (dry gas, wet gas and hydrocarbon liquids). The term ‘phase’ refers to the chemical state of a fluid (e.g., gas, liquid or supercritical). In large-scale modelling of fluid migration, it is usually assumed that a water phase is present. This is true for basin modelling techniques, where the water phase is commonly separated from hydrocarbon phases due to the non-polar nature of hydrocarbon fluid molecules.

#### Fluid properties and buoyancy

The primary driving force responsible for fluid flow in the geological media is buoyancy (Appendix A of Hantschel and Kauerauf^7^), which depends on several factors including fluid and rock properties. Fluid properties are described by Equations of State (EoS), which define the relationship between pressure, volume and temperature. The most well known EoS is the Ideal Gas Equation, however such a simple relationship does not accurately model properties of non-ideal fluids and cannot be used in complex scenarios such as geological basin modelling. There are numerous widely used EoS that describe the relationship between fluid pressure, temperature and volume for non-ideal molecules. The Peng–Robinson 1978 (PR78) and Soave–Redlich–Kwong (SRK) EoS are considered one of the more common models in reservoir engineering to predict the phase-change behaviour of hydrocarbon mixtures in reservoirs. Both EoS are known for their accuracy for multi-phase and multi-component fluids at pressures $< 100$ MPa and temperatures between 300 and 500 $^{\circ }$K, covering the conditions exhibited in most geological basins across the world^7,14,16,43^. We extend application of these EoS to CO_2_, methane and hydrogen as the PR78 and SRK EoS only become unstable when the reduced temperature, $T_{r} < 0.6$, whereby $T_{r} = T/T_{c}$, $T =$ temperature and $T_{c} =$ critical temperature^44^. As $T_{c}$ values of CO$_{2}$, methane and hydrogen are 305.1 K, 190.6 K and 33.2 K, respectively, the corresponding minimum $T_{r}$ values for the PR78 and SRK EoS to remain stable are 183 K for CO$_{2}$, 114 K for methane and 20 K for hydrogen. Hence, the PR78 and SRK EoS remain accurate within the range $0\,^{\circ }C< T_{s} < 20\,^{\circ }C$ and at temperatures exhibited in most geological basins across the world. Other EoS that may be used include the Reidlich-Kwong (RK) EoS, which is suited for small, non-polar and short-chained molecules^12,13,15,19^. Other EoS have been developed specifically for use with hydrocarbons, e.g. volatile, light and black oils^17,18^. Component properties for CO$_{2}$, methane, hydrogen, dry gas, wet gas and hydrocarbon fluids are listed on Table 1.

Whilst EoS may be applied to calculate fluid pressure, temperature and volume, viscosity is another important indicator for phase property characterisations and fluid velocities within rocks and geological basins. Viscosity is often modelled as a quantity dependent only on pressure, temperature, density and the amount of dissolved gas^9^. Whilst advanced theories, such as the friction-theory or free-volume model match laboratory data well, a lack of field data and unknown component parameters (especially for heavy hydrocarbon compounds) make these unsuitable for basin modelling^7^.

Compared to methods that require laboratory or field data, direct approaches such as the empirical Lohrenz–Bray–Clark (LBC) model are simpler to apply in basin modelling scenarios where data is often scarce^12,13,45^. Viscosities can be evaluated very fast due to the simple nature of the LBC-formulas. However, models with lower performance are often not usable in fluid flow simulators. It must also be noted that the LBC-model is based on a polynomial of degree 16. Polynomials of such a high degree are known to easily become numerically unstable and therefore LBC-based models must be evaluated with care^7^. Furthermore, the pressure correction for gases in the LBC method had a tabular formula and was not presented entirely^45^. Using this distinction introduces a discontinuity between the liquid and gas viscosity. A modification to the LBC method may be made by applying the Herning mixing rule^46^. This was validated to successfully model multicomponent and multiphase fluid viscosity using the modified LBC approach for an example using the independent Stiel–Thodos method^13,47^. Hence, this modified LBC method is preferred over the original LBC method^13,45^.

**Figure 6:**
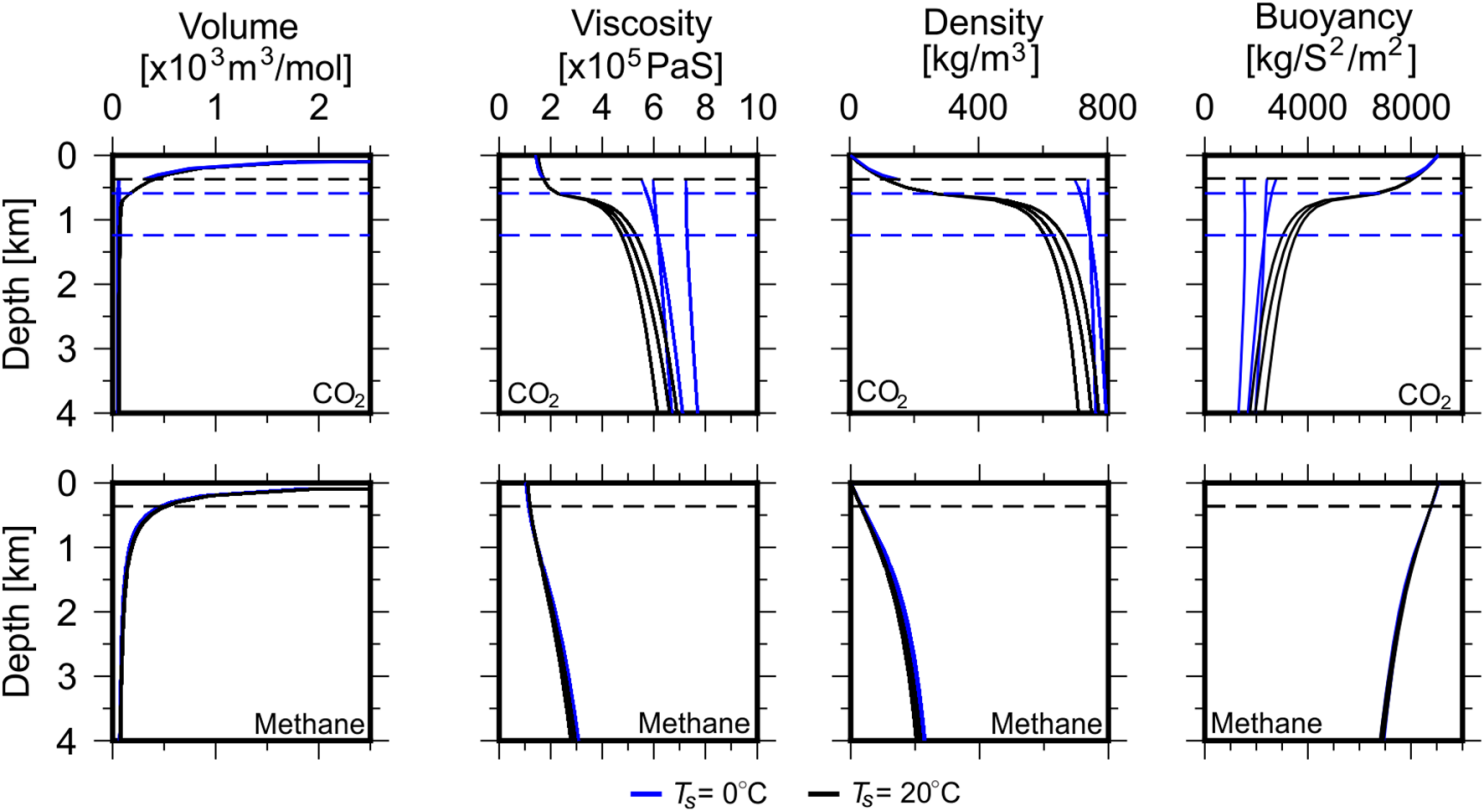
Fluid molar volume, viscosity, density and buoyancy calculated using various Equations of State and modified-LBC method and surface temperature of $20\,^{\circ }$C^12,13^. Blue and black lines represent values calculated using PR78, SRK and Reidlich–Kwong EoS at $T_{s} = 0\,^{\circ }$C and $20\,^{\circ }$C, respectively^14–16,19^. Blue dashed lines represent supercritical-dense liquid and dense liquid–liquid phase transitions for $T_{s} = 0\,^{\circ }$C. Black dashed lines represent gas-supercritical phase transition for $T_{s} = 20\,^{\circ }$C (see Fig. 7). The change from liquid to gas for all fluids is marked by an exponential increase in molar volume and decreases in viscosity and density.

The range of pressure and temperature (PT) paths in in Fig. 5 defines an area of possible PT points in arbitrary sedimentary basins. To simulate generic geological conditions, a geothermal gradient of 25 $^{\circ }$C/km and PT gradient for normal conditions (0.5 MPa/K) was applied (Fig.  5) to calculate the molar volume and density of CO$_{2}$ and methane by solving the PR78, SRK and RK EoS^7,12,13^. The modified-LBC method was used to calculate fluid viscosity (Fig.  6^13^).

**Figure 7:**
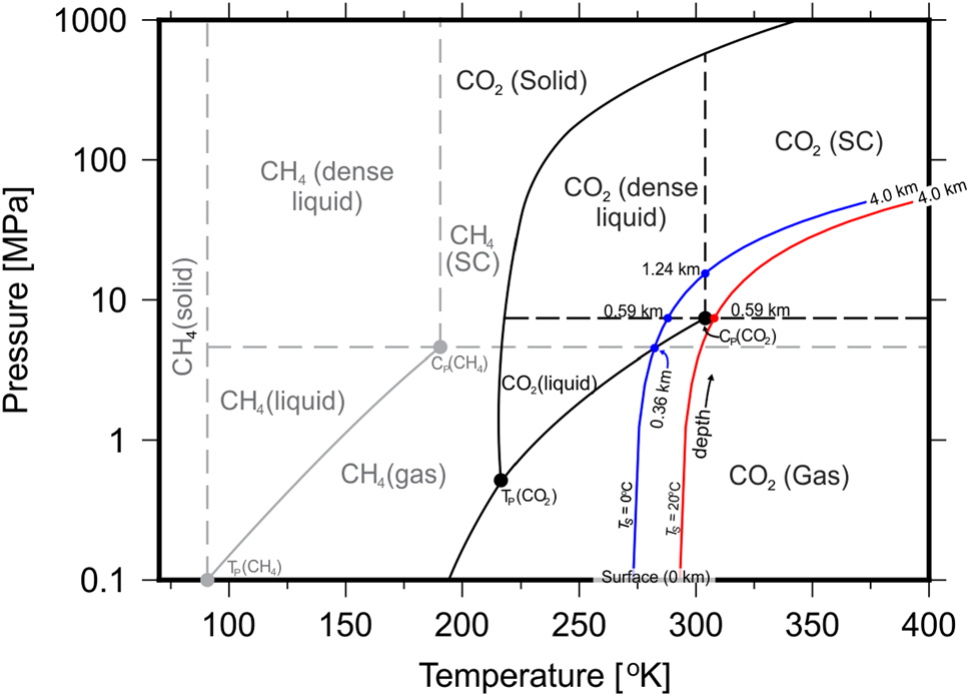
Phase diagram for CO$_{2}$, methane calculated at normal geological conditions and geothermal gradient $= 25\,^{\circ }$C/km. Blue and red lines represent PT paths calculated at $T_{s}$ = $0\,^{\circ }$C and $20\,^{\circ }$C, respectively. Black lines and points represent phase diagram for CO$_{2}$. grey lines and points represent the phase diagram for methane. $T_{p} =$ triple point, $C_{p} =$ critical point.

The transition between phases (solid, dense liquid, liquid, gas and supercritical fluid) is controlled by a fluid’s pressure and temperature relative to its critical pressure, $P_{c}$, and temperature, $T_{c}$. Figure 7 shows phase diagrams of CO$_{2}$ and methane with PT paths for $T_{s} = 0\,^{\circ }$C and $20\,^{\circ }$C. For CO$_{2}$, $P_{c} = 7.3773$ MPa and $T_{c} = 30.98\,^{\circ }$C and is encountered at 0.59 km under normal geological conditions. When $T_{s} < 20\,^{\circ }$C, CO$_{2}$ undergoes three phase transitions at 0.36 km (gas–liquid), 0.59 km (liquid-dense liquid) and 1.24 km (dense liquid-supercritical). However, when $T_{s} \ge 20\,^{\circ }$C, only one phase transition occurs at 0.59 km (gas-supercritical). For methane, all conditions within the Earth irrespective of $T_{s}$ place PT paths within the gas-supercritical region and only one phase transition occurs at 0.36 km.

### Porosity, permeability and saturation

**Figure 8:**
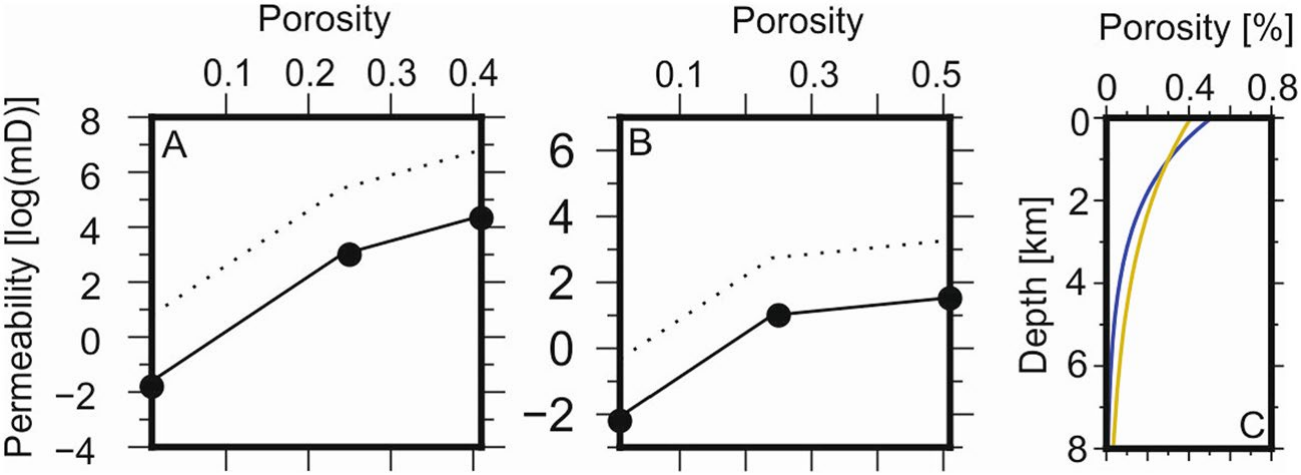
Upscaled rock permeability and porosity. Values from Table 2 (black circles) are used to calculate rock permeability between maximum (depositional) porosity and zero^7^. A = sandstone, B = carbonate. Solid and dotted lines represent vertical and horizontal permeability, respectively. C = porosity-depth relationship calculated using Athy’s relation^48^. Gold and blue lines represent sandstone and carbonate, respectively.

The velocity of fluids in rocks (e.g. sandstone and carbonate) depend on a number of factors, including properties of fluids (Table 1) and rocks (Fig. 8). Another important parameter is porosity, $\phi$, and is defined as the ratio of free pore space to rock volume. $\phi$ controls the volume between grains in a sedimentary rock which may hold fluids and reduces as with compaction. The variation of $\phi$ with depth must be considered when evaluating fluid velocities through rocks. Athy’s relation parametrises $\phi$ using:

12 $$\begin{aligned} \phi = \phi _{0}e^{-z/k} \end{aligned}$$

whereby $\phi _{0}$ is the maximum (depositional) porosity and *k* is the Athy compaction parameter (Table 2^48^).

The flow of fluids through rocks generally involves more than a single phase. The ability of one fluid to flow through a rock or reservoir is affected by the presence of other fluids. Relative permeability, $k_{rp}$ describes multiphase flow in reservoirs as the ratio of the effective permeability of a fluid to the absolute permeability of the rock, $k_{rock}$. The effective permeability is a relative measure of conductance of the porous medium for one fluid phase in the presence of other fluid phases.

Approximating $k_{rock}$ by applying various relationships between porosity, $\phi$ and log($k_{rock}$) is well established and may be performed using several methods and applying appropriate upscaling. These include the multipoint method^7^ or revised Kozeny-Carman relation^49^ for practical use in basin modelling. The multipoint method describes the relationship between $\phi$ and log($k_{rock}$) as a piecewise linear function (Fig.  8). A compilation of porosity-permeability data points for different lithologies are tabulated in Appendix A of Hantschel and Kauerauf^7^. The revised Kozeny–Carman relation^49^ describes $k_{rock}$ as a function of porosity, lithology-dependent specific area and scaling factor. The multipoint and revised Kozeny–Carman relation yield similar values for $k_{rock}(\phi )$ (see Figure 2.16 in Hantschel and Kauerauf^7^), however as the latter is not suitable for carbonate rocks we restrict our analyses to the multipoint method. Combination with Eq. (12) allows computation of $k_{rock}$ as function of depth and lithology-dependent initial porosity and compaction parameters.

Vertical and horizontal rock permeabilities for use in basin modelling may be calculated from hand-specimen measurements by using appropriate anisotropy and upscaling factors. Anisotropy is the ratio of horizontal and vertical permeability and is dependent on rock type. Basin scale values for horizontal and vertical permeabilities are calculated from hand specimen values multiplied a horizontal and vertical upscaling factor, respectively. The resulting higher values for larger scales are caused by macro-fractures, inhomogeneities and permeable inclusions^7^. Here, we apply typical upscaling factors of 1 (vertical) and 50 (horizontal). However, greater upscaling factors (e.g. vertical = 10 and horizontal = 500) to basin scale elements with lengths greater $>50$ m are reported in the literature for sandstones^50^.

Numerous models for estimating relative permeability have been proposed over the last half century. Most relative permeability models assume relative permeability to be a function of connate water saturation ($S_{w}$) and connate gas saturation ($S_{g}$) respectively^51^. The relative permeability of any fluid is zero below its critical saturation, where it becomes immobile. Saturations are for often rescaled into normalised or effective saturations ($S_{e}$) which map the saturation interval between the connate and the critical saturation to an interval of $0< S_{e} < 1$. The relationships between normalised saturations of water, $S_{we}$, gas, $S_{ge}$ and oil–gas, $S_{goe}$, are as follows:

13 $$\begin{aligned} \begin{array}{lll} S_{we} = (S_{w} - S_{wc})/(1 - S_{wc} - S_{oc}) &{}\quad {\text{ for }} &{} k_{rw}, k_{row} \\ S_{goe} = S_{g}/(1 - S_{wc}) &{}\quad {\text{ for }} &{} k_{rog} \\ S_{ge} = (S_{g} - S_{gc})/(1 - S_{wc} - S_{gc}) &{}\quad {\text{ for }} &{} k_{rg} \\ \end{array} \end{aligned}$$

whereby $k_{rw}$, $k_{row}$, $k_{rog}$ and $k_{rg}$ are the relative permeabilities of water, oil–water, oil–gas and gas components respectively. $k_{rw}$ and $k_{rog}$ represent water-liquid flow whilst $k_{rg}$ and $k_{rog}$ represent liquid-vapour flow, respectively. Hence, $k_{rw} = f(S_{w})$, $k_{rg} = f(S_{g})$, $k_{row} = f(S_{w})$ and $k_{rog} = f(S_{g})$. Assuming that phases do not interact during flow, the flow of each phase can be treated as if the other phases are part of the solid rock matrix^51^. In this case, the relative permeability of oil, $k_{ro} = k_{row}k_{rog}$. The quadratic relationship of Hantschel and Kauerauf^7^ may therefore be applied to determine relative permeabilities from normalised effective saturation values. We use this relationship to approximate the flow of the fluids (Table 1) in sandstones and carbonates.

### Water saturation from porosity

Determining water saturation, $S_{w}$, is an extremely challenging petrophysical calculation to perform. $S_{w}$ is used to quantify the hydrocarbon saturation, $(1 - S_{w})$ and must be evaluated in basin modelling. Complexities arise because there are a number of independent approaches that can be used to calculate $S_{w}$. In wellbores, the following methods can be used to determine $S_{w}$:

- $S_{w}$ calulations from resistivity logs and by application of a model relating $S_{w}$ to porosity, $\phi$, connate water resistivity and lithology-dependent electrical properties.
- $S_{w}$ calculations from laboratory capillary pressure and saturation ($P_{cap}/S_{w}$) measurements by application of a model relating $S_{w}$ to various rock, fluid properties and height above the free-water level.
- $S_{w}$ calculations using oil-based mud (OBM)-core-plug Dean–Stark–water-volume determinations.
- Combinations of these methods

The amount of data that is available often dictates which method to use to determine $S_{w}$. For the purposes of basin modelling, which requires the application of generalised lithologies and fluid types, $S_{w}$ must be calculated from data input by the user, e.g. rock type, fluid type and depth. Laboratory-based methods for measuring $S_{w}$ are not suitable for this work, as we are concerned with the properties of generalised rock types and fluids on the basin-scale.

**Figure 9:**
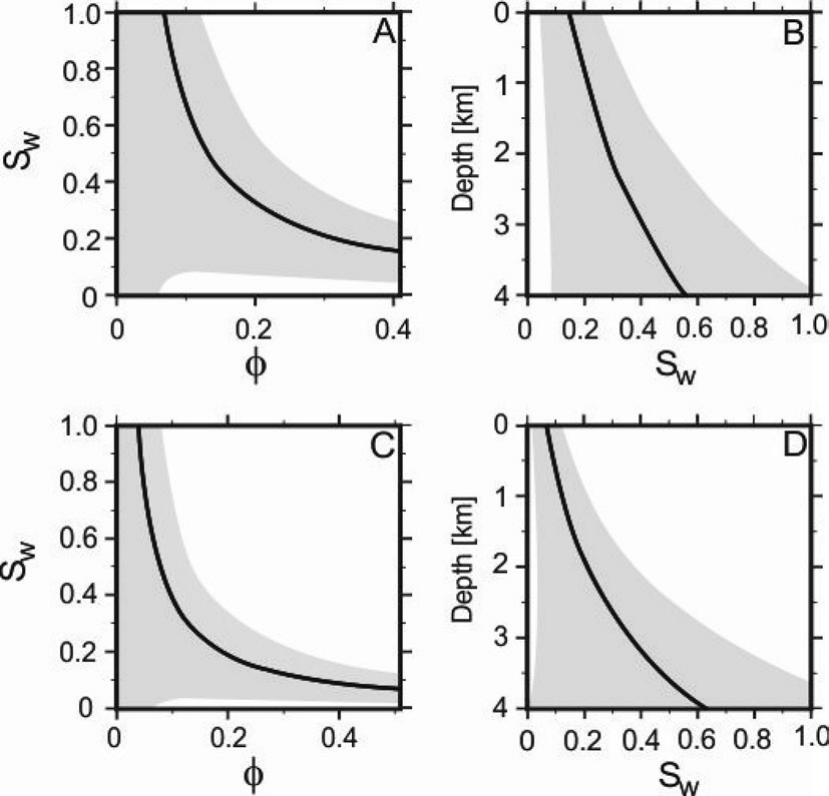
Connate water saturation ($S_{w}$) as a function of porosity and depth calculated using parameters for sandstone and carbonate and Athy’s relation^7,48,53^. (**A**,**B** )= sandstone, (**C**,**D**) = carbonate. Grey regions represent uncertainties calculated using upper and lower bounds of experimental constants for Eq. (14)^53^. Uncertainties beyond depths of 2 km become significant, hence we do not extend this method beyond this depth.

The results of early experiments indicated that the product of $\phi$ and $S_{w}$ is constant^52^. The magnitude of this constant was shown to be a related to rock type and indirectly to permeability, *k*. Better quality rocks were found to correspond with low constant values. Extensive analysis of core data and petrophysical estimates of porosity and irreducible water saturation, from all types of reservoirs worldwide, suggests that this relation is a unique solution to a more general equation:

14 $$\begin{aligned} \phi ^{Q} \times S_{w} = C \end{aligned}$$

whereby the value of the power function *Q* ranges from 0.8 to 1.3, with many reservoirs close to 1.0. For sandstones, $0.02< C < 0.10$ and $0.005< C < 0.06$ for carbonates. The values of *Q* and constant *C* can be easily derived by plotting log$\phi$ against log$S_{w}$. The gradient of this linear plot $= Q$. Projection of the straight line against $\phi = 1.0$ gives the value of the constant, *C*^52,53^. Figure 9 shows the relationship between $S_{w}$ and $\phi$ calculated using Eq. (14) and depth from Eq. (12). Uncertainties may be calculated using the upper and lower bounds of experimental constants and exponent terms from laboratory measurement^53^. Uncertainty increases significantly as porosity is reduced through compaction ($\phi < 5\%$) and with depth. Uncertainties increase significantly below 2 km and cover almost the entire range of possible $S_{w}$ values by 4 km depth. Hence, we recommend that application of this methodology should be limited to depths above 2 km. Future laboratory measurements of coefficients of Eq. (14) may lower uncertainties and increase model robustness beneath 2 km.

## Data Availability

All data generated or analysed during this study are included in this published article.

## References

[1] Geoscience Australia. *Australia’s Energy Commodity Resources 2021*. https://www.ga.gov.au/digital-publication/aecr2021 (2021).

[2] U.S. Energy Information Administration. Natural gas explained, Where our natural gas comes from. https://www.eia.gov/energyexplained/natural-gas/where-our-natural-gas-comes-from.php (2021).

[3] Her Majesty’s Government. *Net Zero Strategy: Build Back Greener* (2021).

[4] Klima, E. F. Lov om klima. https://www.retsinformation.dk/eli/lta/2020/965 (2020).

[5] British Petroleum. *BP Sets Ambition for Net Zero by 2050, Fundamentally Changing Organisation to Deliver*. https://www.bp.com/en/global/corporate/news-and-insights/press-releases/bernard-looney-announces-new-ambition-for-bp.html (BP Press, 2020).

[6] Shell, R. D. Shell accelerates drive for net-zero emissions with customer-first strategy. https://www.shell.com/media/news-and-media-releases/2021/shell-accelerates-drive-for-net-zero-emissions-with-customer-first-strategy.html (2021).

[7] Hantschel T, Kauerauf A (2009). Fundamentals of Basin and Petroleum Systems Modeling.

[8] Ihrig C, Unnithan V, Lohmann G, Grosfeld K, Wolf-Gladrow D, Wegner A, Notholt J, Unnithan V (2013). Feasibility study of using a petroleum systems modelling software to evaluate basin scale pressure evolution associated with CO_2_ storage. Geoengineering.

[9] Danesh, A. PVT and phase behaviour of petroleum reservoir fluids. In *Number 47 in Developments in Petroleum Science’* (Elsevier, 1998).

[10] IES PetroMod®. *Petroleum Systems Modeling Software, Release 10.0* (2007).

[11] Reid RC, Prausnitz J, Poling B (1987). The Properties of Gases and Liquids.

[12] Bell, C. *chemicals: Chemical properties component of Chemical Engineering Design Library (ChEDL)*. https://github.com/CalebBell/chemicals (2021a).

[13] Bell, C. *Thermo: Chemical properties component of Chemical Engineering Design Library (ChEDL)*. https://github.com/CalebBell/thermo (2021b).

[14] Peng, D.-Y. & Robinson, D. B. The characterization of the heptanes and heavier fractions for the GPA Peng–Robinson programs. In *Gas Processors Association* (Tulsa, Oklahoma, 1978).

[15] Poling B (2000). The Properties of Gases and Liquids.

[16] Soave G (1972). Equilibrium constants from a modified Redlich–Kwong equation of state. Chem. Eng. Sci..

[17] Twu CH, Coon JE, Cunningham JR (1995). A new generalized alpha function for a cubic equation of state Part 1. Peng–Robinson equation. Fluid Phase Equilib..

[18] Twu CH, Coon JE, Cunningham JR (1995). A new generalized alpha function for a cubic equation of state Part 2. Redlich–Kwong equation. Fluid Phase Equilib..

[19] Walas S (1985). Phase Equilibria in Chemical Engineering.

[20] Sylta Ø (2004). Hydrocarbon Migration Modelling and Exploration Risk.

[21] Busch A, Alles S, Gensterblum Y, Prinz D, Dewhurst DN, Raven MD, Stanjek H, Krooss BM (2008). Carbon dioxide storage potential of shales. Int. J. Greenh. Gas Control.

[22] Intergovernmental Panel on Climate Change. *IPCC Special Report on Carbon Capture and Stoage*, Vol. 58 (2005).

[23] Smerdon JE, Pollack HN, Cermak V, Enz JW, Kresl M, Safanda J, Wehmiller JF (2004). Air-ground temperature coupling and subsurface propagation of annual temperature signals. J. Geophys. Res. D Atmos..

[24] Zhang ZX, Wang GX, Massarotto P, Rudolph V (2006). Optimization of pipeline transport for CO_2_ sequestration. Energy Convers. Manag..

[25] Krevor S, Blunt M, Benson S, Pentland C, Reynolds C, Al-Menhali A, Niu B (2015). Capillary trapping for geologic carbon dioxide storage—From pore scale physics to field scale implications. Int. J. Greenh. Gas Control.

[26] Lesperance M, Smerdon JE, Beltrami H (2010). Propagation of linear surface air temperature trends into the terrestrial subsurface. J. Geophys. Res. Atmos..

[27] Kurylyk BL, MacQuarrie KTB (2014). A new analytical solution for assessing climate change impactson subsurface temperature. Hydrol. Process..

[28] Taniguchi M, Uemura T, Jago-on K (2007). Combined effects of urbanization and global warming on subsurface temperature in four Asian cities. Vadose Zone J..

[29] Westaway R, Younger PL (2016). Unravelling the relative contributions of climate change and ground disturbance to subsurface temperature perturbations: Case studies from Tyneside, UK. Geothermics.

[30] Nelson JS, Simmons EC (1995). Diffusion of methane and ethane through the reservoir cap rock: Implications for the timing and duration of catagenesis. Am. Assoc. Pet. Geol. Bull..

[31] Chapoy A, Burgass R, Terrigeol A, Coquelet C (2015). Water content of CO_2_-rich mixtures: Measurements and modeling using the cubic-plus-association equation of state. J. Nat. Gas Eng..

[32] Hu J, Duan Z, Zhu C, Chou IM (2007). PVTx properties of the CO_2_–H_2_O and CO_2_–H_2_O–NaCl systems below 647 K: Assessment of experimental data and thermodynamic models. Chem. Geol..

[33] Valtz A, Chapoy A, Coquelet C, Paricaud P, Richon D (2004). Vapour-liquid equilibria in the carbon dioxide–water system, measurement and modelling from 278.2 to 318.2 K. Fluid Phase Equilib,.

[34] Wang T, El Ahmar E, Coquelet C, Kontogeorgis GM (2018). Improvement of the PR-CPA equation of state for modelling of acid gases solubilities in aqueous alkanolamine solutions. Fluid Phase Equilib..

[35] Gilmore KA, Sahu CK, Benham GP, Neufeld JA, Bickle MJ (2022). Leakage dynamics of fault zones: Experimental and analytical study with application to CO_2_ storage. J. Fluid Mech..

[36] Sun Q, Wu S, Lu F, Yuan S (2010). Polygonal faults and their implications for hydrocarbon reservoirs in the southern Qiongdongnan Basin, South China Sea. J. Asian Earth Sci..

[37] Peacock, B. *Natural Hydrogen Exploration ‘Boom’ Snaps Up One Third of South Australia*. https://www.pv-magazine.com/2022/02/02/natural-hydrogen-exploration-boom-snaps-up-one-third-of-south-australia/ (2022).

[38] Lefeuvre N, Truche L, Donzé FV, Ducoux M, Barré G, Fakoury RA, Calassou S, Gaucher EC (2021). Native H2 exploration in the western pyrenean foothills. Geochem. Geophys. Geosyst..

[39] Prinzhofer A, Tahara Cissé CS, Diallo AB (2018). Discovery of a large accumulation of natural hydrogen in Bourakebougou (Mali). Int. J. Hydrog. Energy.

[40] Rahbari A, Brenkman J, Hens R, Ramdin M, Van Den Broeke LJ, Schoon R, Henkes R, Moultos OA, Vlugt TJ (2019). Solubility of water in hydrogen at high pressures: A molecular simulation study. J. Chem. Eng. Data.

[41] Levorsen A (1967). Geology of Petroleum.

[42] Selley R (1985). Elements of Petroleum Geology.

[43] Al-Kindi I, Babadagli T (2021). Revisiting Kelvin equation and Peng–Robinson equation of state for accurate modeling of hydrocarbon phase behavior in nano capillaries. Sci. Rep..

[44] Ghanbari M, Ahmadi M, Lashanizadegan A (2017). A comparison between Peng-Robinson and Soave–Redlich–Kwong cubic equations of state from modification perspective. Cryogenics.

[45] Lohrenz J, Bray BG, Clark CR (1964). Calculating viscosities of reservoir fluids from their compositions. J. Pet. Technol..

[46] Herning F (1935). Beitrag zur Berechnung der Zähigkeit technischer Gasgemische aus den Zähigkeitswerten der Einzelbestandteile.

[47] Stiel LI, Thodos G (1961). The viscosity of nonpolar gases at normal pressures. AIChE J..

[48] Athy LF (1930). Density, porosity, and compaction of sedimentary rocks. AAPG Bull..

[49] Ungerer P, Burrus J, Doligez B, Chenet P, Bessis F (1990). Basin evaluation by integrated two-dimensional modeling of heat transfer, fluid flow, hydrocarbon gerneration and migration. AAPG Bull..

[50] Schulze-Makuch D, Carlson D, Cherkauer D, Malik P (1999). Scale dependency of hydraulic conductivity in heterogeneous media. Ground Water.

[51] Aziz K, Settari A (1979). Petroleum Reservoir Simulation.

[52] Buckles R (1965). Correlating and averaging connate water saturation data. J. Can. Pet. Technol..

[53] Holmes M, Holmes A, Holmes D (2009). Relationship between porosity and water saturation: Methodology to distinguish mobile from capillary bound water two different rock types. AAPG Annu. Conv..

